# Association of Functional Impairments and Co-Morbid Conditions with Driving Performance among Cognitively Normal Older Adults

**DOI:** 10.1371/journal.pone.0167751

**Published:** 2016-12-22

**Authors:** David B. Carr, Peggy P. Barco, Ganesh M. Babulal, Sarah H. Stout, Anne M. Johnson, Chengjie Xiong, John C. Morris, Catherine M. Roe

**Affiliations:** 1 Division of Geriatrics and Nutritional Science, Division of Neurorehabilitation, Washington University in St. Louis, The Rehabilitation Institute of St. Louis (TRISL), Parc Provence, St. Louis, MO, United States of America; 2 Program in Occupational Therapy, Washington University in St. Louis, St. Louis, MO, United States of America; 3 Department of Neurology, Washington University in St. Louis, St. Louis, MO, United States of America; 4 Knight Alzheimer’s Disease Research Center, Washington University in St. Louis, St. Louis, MO, United States of America; 5 Center for Clinical Studies, Washington University in St. Louis, St. Louis, MO, United States of America; 6 Division of Biostatistics, Washington University in St. Louis, St. Louis, MO, United States of America; 7 The Center for Aging, Memory and Aging Project, Washington University in St. Louis, St. Louis, MO, United States of America; Nathan S Kline Institute, UNITED STATES

## Abstract

**Objectives:**

To examine the relationship between key functional impairments, co-morbid conditions and driving performance in a sample of cognitively normal older adults.

**Design:**

Prospective observational study

**Setting:**

The Knight Alzheimer’s Disease Research Center, Washington University at St. Louis

**Participants:**

Individuals with normal cognition, 64.9 to 88.2 years old (N = 129), with a valid driver’s license, who were currently driving at least once per week, and who had participated in longitudinal studies at the Knight Alzheimer’s Disease Research Center

**Measurements:**

Static visual acuity, contrast sensitivity, physical frailty measures, motor skills, total medical conditions, and the modified Washington University Road Test.

**Results:**

When controlling for age, race, gender, APOE, and education the total number of medical conditions was unassociated with both road test scores (pass vs. marginal + fail) and the total driver error count. There were marginal associations of our measure of physical frailty (p = 0.06) and contrast sensitivity score (p = 0.06) with total driving error count.

**Conclusion:**

Future research that focuses on older adults and driving should consider adopting measures of physical frailty and contrast sensitivity, especially in samples that may have a propensity for disease impacting visual and/or physical function (e.g. osteoarthritis, Parkinson’s, eye disorders, advanced age >80 years, etc.).

## Introduction

Driving an automobile is a crucial instrumental activity of daily living and it can become increasingly difficult with age. Approximately 200,000 of the 30 million drivers 65 years of age or older in the United States are injured in motor vehicle crashes each year [1] and there were over 4,000 motor vehicle deaths for those aged 70 years or older in 2014 [2]. Even though many older persons self-restrict their driving to compensate for age-related changes and diseases [3], crash rates per mile traveled start increasing for drivers at age 70 and older and are highest after age 85 [1]. Furthermore, two longitudinal driving studies that included samples of cognitively intact older adults have revealed deterioration in driving performance over time on standardized performance based road tests [4, 5].

The etiology for this decline in driving performance is unclear. Our study group recently published on a sample of 129 cognitively normal older adults and found an increased number of driving errors associated with increasing levels of molecular biomarkers for Alzheimer disease (AD), suggesting a possible functional correlate of preclinical AD [6]. However, other causes should also be considered since functional impairments in other key domains required for driving (e.g. vision, motor ability) and/or additional co-morbid conditions (e.g. diabetes, heart disease) could impair driving performance via other mechanisms.

Impairments in vision and neuromuscular strength and speed have been linked to crash risk for older adults [7]. Common age-related eye diseases such as macular degeneration, cataracts and glaucoma, may result in subsequent loss of contrast sensitivity and restricted visual fields, which have been associated with impaired driving [8, 9]. Reduced neck rotation, orthostatic drop in blood pressure, slow foot reaction time and a history of a fall have been associated with increase crash risk [10–12]. Use of certain medications, including benzodiazepines, opioid analgesics, alcohol, muscle relaxants, sedating antihistamines and antidepressants, is also linked to increased risk [13, 14]. A myriad of medical conditions associated with impaired driving performance and increased crash risk have also been the subject of recent reviews [15, 16].

In this study, we examined the relationship between key functional impairments, co-morbid conditions and driving performance in a sample of cognitively normal older adults. We tested whether the presence of functional impairment and comorbid conditions were associated with road test errors. We hypothesized that multiple medications and medical conditions or the presence of visual and/or physical functional impairment would be associated with worsening driving performance.

## Materials and Methods

### Design

Participants with normal cognition (Clinical Dementia Rating [CDR] = 0) [17], aged 65 years and older, with a valid driver’s license, and who were currently driving at least once per week, were recruited for this cross-sectional study (AG043434) from participants in longitudinal studies at the Knight Alzheimer’s Disease Research Center (ADRC). At baseline, participants took part in annual clinical and psychometric assessments performed by the clinical core in the Knight ADRC. This was followed by additional functional based measures associated with impaired driving performance and then a standardized performance based road test. Written informed consent was obtained from all participants. This study was approved by the Washington University Human Studies Committee.

### Clinical and psychometric assessments

A CDR is derived by experienced clinicians who synthesize information obtained from semi-structured interviews with the participant and separately with a collateral source that has familiarity with the participant. The CDR is derived in accordance with a standard scoring algorithm and only those CDR = 0 (cognitively normal) were recruited for this study.

### Measurement of functional domains

#### Vision

The participant was assessed for far visual acuity by Early Treatment of Diabetic Retinopathy Study (ETDRS) Chart [18]. Contrast sensitivity was tested using the Pelli-Robson contrast sensitivity chart [19].

#### Physical frailty

Four measures of the 9-item Physical Performance Test PPT [20] were completed annually on participants and include timed ability to pick up a coin, timed 50-foot walk, time to perform 5 chair stands and balance testing. These four measures were combined for an overall frailty score. Only the time for the full tandem stance was selected for the balance measure, since this was the only balance subscore where any impairment was documented.

#### Motor skills

The clinician scored motor examination measures using the Unified Parkinson’s Disease Rating Scale: Part III [21], which uses a Likert scale (0–4, with higher scores indicating more impairment) to assess speech, facial expression, tremor at rest and with action, rigidity, finger taps, hand movements, leg agility, chair stands, posture, postural stability and gait.

### Measurement of co-morbid conditions

#### Medical conditions

A total count of medical conditions was calculated at the baseline clinical assessment prior to the driving test by summing the number of medical conditions reported by the participant during the annual clinical assessment. The presence/history of the following active medical conditions in the past five years was assessed: myocardial infarction, cardiac arrest, atrial fibrillation, coronary artery disease, s/p coronary artery bypass graft, s/p pacemaker placement, congestive heart failure, stroke, transient ischemic attack (TIA), Parkinson’s disease, seizures, traumatic brain injury (TBI), hypertension, hypercholesterolemia, diabetes, B12 deficiency, thyroid disease, incontinence, depression, substance abuse, and psychiatric disorders not otherwise specified (NOS).

#### Medications

A total count of routine medications taken by each participant at the time of the baseline yearly clinical assessment prior to the driving assessment was obtained from the participant. Medications examined were antihypertensives (e.g. angiotensin converting enzyme Inhibitors, calcium channel blockers, angiotensin receptor blockers, benzodiazepines, statins, bladder agents, diabetic agents, tricyclic antidepressants, antiepileptic agents, hypnotics, antipsychotics, selective serotonin reuptake inhibitors, H2 receptor antagonists, Parkinson’s agents).

#### Performance based road test

The 12-mile, modified Washington University Road Test (mWURT), takes about an hour to complete and is scored using both ordinal methodology (e.g. pass, marginal, or fail) [22, 23]and a quantitative count of total driving errors or abnormal behaviors (e.g. the Record of Driving Errors)[24]. The course begins in a closed parking lot so that the participant becomes familiar with the study car, a 4-door sedan, and then proceeds to a community in-traffic route which includes unprotected left hand turns, in addition to complex intersections and lane merges. Following directions delivered by an occupational therapy/driving rehabilitation specialist (OTR/DRS) sitting in the front seat, the participant drives throughout the mWURT route. A portion of the road course is self-directed driving. This aspect of the evaluation requires a participant to locate a specific store, find the entrance, park, and navigate out of the parking lot. The front-seat OTR/DRS can take control of the wheel if needed or apply a passenger-side brake. For the purposes of this analysis, road test performance was treated as a dichotomous outcome (i.e., pass ratings compared to marginal and fail ratings combined) [25]. The average time between the baseline annual assessment and the performance-based road test was 4.2 months.

#### Statistical analyses

Analyses were performed using SAS, version 6.0 (SAS Institute, Inc., Cary, NC). Logistic regression (for road test rating) and linear models (total number of road test errors), after adjusting for age, education, gender, race, and apolipoprotein E4 (*APOE4)*, were used to test whether road test outcomes differed for the presence or absence of each functional domain measure or as a function of our co-morbidity index values (i.e. total number of medical conditions or medications).

## Results

Individuals aged 64.9 years to 88.2 years (N = 129) met inclusion criteria (Table 1) and had the majority of clinical information available for analysis. Participants were of advanced age (avg. 73 years, predominantly Caucasian (~90%), of equal gender, highly educated, and with an expected normal mental status screen (average MMSE = 29). Twenty-nine % of the sample had at least one positive ApoE4 allele. Table 2 provides averages of our non-cognitive co-morbid and functional measures, which include total number of medical conditions, medication, physical frailty score, UPDRS score, visual acuity, and contrast sensitivity. However, only a subset of the sample had physical performance data recorded (N = 84), since participants in the Adult Children study did not have these annual measures (Table 2). When controlling for age, race, gender, APOE, and education the total number of medical conditions and medications were unassociated with both our qualitative road test scores (pass vs. marginal + fail) using logistic regression and the total driver error count, using linear regression. There were marginal associations of our measure of physical frailty (p = 0.06) and contrast sensitivity score (p = 0.06) with total driving error count (Tables 3 and 4).

**Table 1:** Demographics (N = 129).

Characteristics	Mean/(SD)
Age (years)	72.9 (4.9)
Minority (African-American)	9.3%
Gender (% Women)	53.5%
Education, mean years, (SD)	16.1(2.59)
MMSE, mean (SD)*	29.4(0.9)
Clinical Dementia Rating = 0, (%)	100
APOE4 presence of one allele	29%

Abbreviations: *APOE*4 = apolipoprotein E; MMSE, Mini-Mental State Exam

* MMSE scores range from 0 (worst performance) to 30 (best performance)

**Table 2:** Summary of Functional Assessment/Co-morbidities (N = 129).

Co-morbid/Functional Conditions	Mean (SD)
Total Number of Medical Conditions	1.5 (1.2)
Total Number of Medications	.8 (1)
Physical Frailty Sum Score (secs)/N = 84	12.4 (1.8)
UPDRS Total Score	6.1 (12.5)
Far Visual Acuity OU (mean)	26.1 (7.6)
Contrast Sensitivity (logMar)	1.6 (.1)

**Table 3:** Regression Analysis of Physical Frailty Measure with Road Test Total Error Count.

Variable	Coefficient Estimate	Standard Error	t Value	Pr>|t|
**Intercept**	-29.59298323	14.40705995	-2.05	0.0434
**Physical Frailty Measure**	-0.62810913	0.32952665	-1.91	0.0604
**Education**	0.11268732	0.22447777	0.50	0.6171
**Gender**	1.28776597	1.08542959	1.19	0.2391
**Age**	0.38069143	0.11607072	3.28	0.0016
**Apoe4**	-1.41015024	1.16837862	-1.21	0.2312
**Race**	3.11646881	2.18982702	1.42	0.1587

**Table 4:** Regression Analysis of Contrast Sensitivity Measure with Road Test Total Error Count.

Variable	Coefficient Estimate	Standard Error	t Value	Pr>|t|
**Intercept**	-11.54753243	13.78748131	-0.84	0.4039
**Contrast Sensitivity(logMars)**	-7.86575693	4.24549330	-1.85	0.0663
**Education(yrs)**	0.10691075	0.16769699	0.64	0.5250
**Gender**	1.22280783	0.83625068	1.46	0.1462
**Age(years)**	0.31142642	0.09628342	3.23	0.0016
**Apoe4+**	-1.32699195	0.89329747	-1.49	0.1400
**Race**	1.37946810	1.41807459	0.97	0.3326

## Discussion

We did not find evidence for an association between our comorbidity measures and road test performance (total number of driving errors or qualitative ratings). This may not be surprising given the voluntary nature of our cohort, which recruits healthy older adults in the community for longitudinal studies on memory and aging. The nature of our cohort, as reflected in Table 2, suggests a very low prevalence of co-morbid conditions and scores in the normal range on our functional measures.

We did find a marginal relationship between both physical frailty and contrast sensitivity with total driving error count. There could be several explanations for these findings. Motor decline has been noted in some studies on aging and driving and our driving sample may have captured a cohort of drivers with subtle, but significant, age-related sarcopenia and/or muscle weakness impacting driving performance [25, 26]. Another explanation for this potential relationship is preclinical AD itself. The physical frailty measures of time and reduced speed of processing might have been impacted by drivers with abnormal AD biomarkers. Contrast sensitivity has been associated with impaired driving performance in older adults. However, the Pelli-Robson Chart may not be as sensitive as other measures of contrast sensitivity [27]. Thus, we may have missed an opportunity for a more accurate measurement of this construct. In addition, the road course was done during the day and did not consistently present opportunities to challenge participants in low contrast settings. Finally, these marginal relationships may be spurious findings.

There were several limitations to this study. Static (non-dynamic/without movement) visual acuity is usually measured in performance based road test studies due to state licensing requirements. However, there is typically no correlation with this measure and driving performance [28]. Thus, the lack of relationship with this measure and driving ability in our sample is not surprising. The performance based road test was not recorded and reviewed by video and was based only on a one hour assessment. It is possible that a video recorded study over a longer period of time and/or a naturalistic study might have captured more errors that would pertain to driving safety. There is a small portion of our course that does require self-directed driving (i.e. strategic level). A course that requires more route planning and self-directed driving might reveal more driving performance concerns.

This research volunteer sample was recruited from the community and was relatively healthy. All 129 participants in this sample had molecular biomarkers studies for AD and this requirement limits the representativeness of the ADRC sample. Although participants are followed longitudinally, this manuscript reports on cross-sectional baseline data collected and those processed to date. These research volunteers may not be representative of the general population. A sample based in a medical setting would likely have more sizable co-morbidities and possibly shown a relationship with driving performance. Our measures of co-morbidity were a simple sum of the number of medications or medical conditions. A more detailed measure of disease severity that includes functional limitations such as the Geriatric Co-morbidity Index, may have revealed a relationship with driving ability [29]. Finally, we did not explore the contribution of cognitive impairment as measured by psychometric test performance in this paper. Stage III Preclinical AD [30] is manifested by impaired cognitive testing and will be the basis of a future study.

## Conclusions

In summary, we did not find evidence in this sample for significant visual, motor or co-morbid conditions impacting driving performance. This may be in part related to the recruitment of healthy volunteers. The marginal relationship we found with physical frailty, contrast sensitivity and driving performance is interesting and may indicate that impairments in muscle strength, coordination, gait and/or balance and vision could still play a role either independently or perhaps along with “cognitive frailty” in preclinical AD, and contribute to driving decrements. Future research that focuses on older adults and driving research should consider adopting measures of physical frailty and contrast sensitivity, especially in samples that may have a propensity for disease impacting physical function (e.g. osteoarthritis, Parkinson’s, eye disorders, advanced age >80 years, etc.).

## Supporting Information

S1 Driving Data Functional Impairment and Comorbid Conditions dataset. (XLSX)
